# Photolytic Mass Loss of Humic Substances Measured with a Quartz Crystal Microbalance

**DOI:** 10.1021/acsearthspacechem.4c00134

**Published:** 2024-07-11

**Authors:** Mingrui Sun, Geoffrey D. Smith

**Affiliations:** Department of Chemistry, University of Georgia, Athens, Georgia 30602, United States

**Keywords:** photolysis mass loss, condensed-phase photolysis, aqueous photolysis, humic substance, secondary organic aerosol

## Abstract

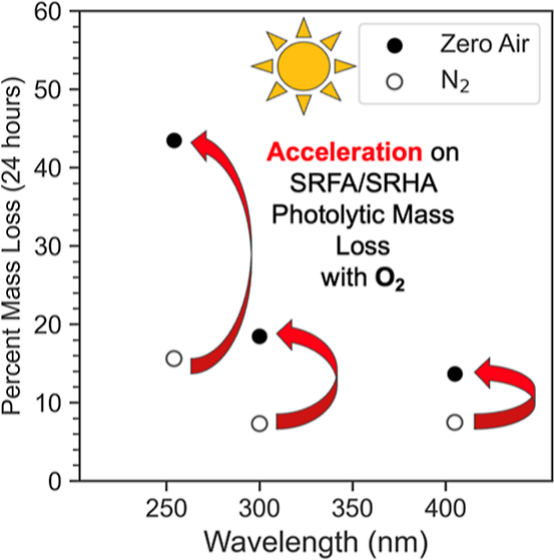

Laboratory studies have shown that photolytic mass loss can be a significant sink for secondary organic aerosol (SOA). Here, we use a quartz crystal microbalance to measure mass loss of Suwannee River Humic Acid (SRHA) and Suwannee River Fulvic Acid (SRFA), surrogates for SOA, exposed to 254, 300, and 405 nm radiation over the course of 24 h. We find that the photolytic mass loss rates of these materials are comparable to those for laboratory-generated limonene and toluene SOA material from the study of Baboomian et al, *ACS Earth Space Chem*. **2020,***4,* 1078. Scaling our results to ambient conditions, we estimate that humic substances in aerosols can lose as much as 8% by mass in the first day of exposure in the atmosphere, equivalent to 0.025% of *J*_NO_2__, the photolysis rate of nitrogen dioxide. By using zero air instead of nitrogen, we also find that the presence of oxygen accelerates the photolytic mass loss rate by a factor of 2 to 4 at all wavelengths suggesting a potential role for reactive oxygen species. UV photolysis of an aqueous SRFA solution demonstrated both photobleaching at UV wavelengths and photoenhancement at visible wavelengths. Ultrahigh-resolution mass spectrometric analysis showed that condensed-phase SRFA photolysis led to decreased intensity in the 100–300 *m*/*z* range while aqueous SRFA photolysis resulted in an increase in intensity in the same range. This work reaffirms that photolytic mass loss is a potentially significant sink for SOA, but only on the time scale of a day or two and demonstrates that SRHA and SRFA are suitable surrogates for atmospheric SOA with respect to photolytic mass loss.

## 1 Introduction

Secondary Organic Aerosols (SOAs) are ubiquitous in the atmosphere, representing a large portion of global submicron particulate matter and having a major impact on climate and human health.^2^ SOAs are generated through the condensation of oxidation products of volatile organic compounds (VOCs). Due to a huge variety of precursor VOCs and oxidants such as OH, O_3_, and NO_X_, SOAs have complex chemical composition.^3^ In the atmosphere, SOA composition can evolve through various aging processes, including photochemistry, photodegradation, gas-particle partitioning, heterogeneous reactions, aqueous-phase processing, and interactions with other atmospheric components such as oxidants or mineral dust. These aging processes alter the size distribution, hygroscopicity, chemical properties, optical properties and the climate impact of SOAs.^4^ Currently, the photodegradation aging processes of SOAs, in particular, are understudied and pose great uncertainty in quantifying the influence of SOAs on climate.^5^

Photodegradation is the process by which SOAs absorb solar radiation and produce smaller volatile compounds, such as CO, CO_2_, CH_4_, acetic acid, and acetone.^6^ This process reduces the mass concentration of SOAs in the atmosphere and is deemed an important but often overlooked sink for SOA.^7^ Previous modeling efforts have shown that including a universal SOA photolysis rate of 0.04% *J*_NO_2__ (photolysis rate of NO_2_) in the model can better predict the concentration of organic aerosol (OA) in the upper and middle troposphere, where it was overestimated compared to field campaign measurements.^6,8^ This rate of SOA photolysis could significantly reduce the lifetime of SOA from 10 days to 3 days.^8^ Better measurements of this photolysis mass loss rate will enable improved representation of OA in global chemical transport models and will enhance our understanding of this process.

Previous studies of the photolytic mass loss of secondary organic aerosols have primarily focused on lab-generated model SOAs from volatile organic compounds (VOCs) such as isoprene, α-pinene, limonene, and toluene.^1,9−11^ The reported photolysis lifetimes of SOAs vary from a few hours to days,^1,9−11^ with differences in the results attributable to four main factors: (1) the optical and chemical properties of SOA generated from different VOC precursors under varying oxidant conditions can differ, (2) experimental conditions such as relative humidity, temperature, and SOA generation conditions may vary significantly between studies, (3) for chamber studies, chamber wall loss can be a sink for SOAs and must be corrected for,^12^ (4) the chemical composition of SOA changes throughout the photolysis process, so the initial photolysis rate measured is not necessarily representative of the photolysis rate integrated over the lifetime of the SOA. Moreover, lab-generated SOAs may not represent ambient SOAs exactly, as only limited precursors have been investigated and the generation process does not necessarily capture the complex interactions present in ambient SOA formation.

In contrast, humic-like substances (HULIS) may offer a more representative proxy for ambient SOAs due to their ubiquity in the environment and their similar complex chemical compositions.^13^ HULIS are complex macromolecular mixtures that share analogous optical and chemical properties with humic or fulvic acids derived from terrestrial and aquatic sources.^13^ Atmospheric HULIS can be formed through secondary processes such as SOA formation or from primary emissions like biomass and coal burning.^14^ Within the atmosphere, HULIS constitute a substantial fraction of Water-Soluble Organic Carbon (WSOC) (between 20 and 50% by weight) and play a crucial role in aerosol Cloud Condensation Nuclei (CCN) activity.^13,15,16^ Furthermore, HULIS exhibit strong UV light absorption, similar to brown carbon (BrC), and are photoactive, participating in various atmospheric photochemical processes.^17^ Although atmospheric HULIS generally consist of smaller molecules than aquatic humic or fulvic acids, the use of aquatic humic standards such as Suwannee River Humic Acid (SRHA) or Fulvic Acid (SRFA) as proxies for ambient secondary aerosols or atmospheric HULIS is common.^13,18^ In addition, humic standards can be purchased, whereas lab-generated SOA must be created, sometimes requiring specialized equipment.

Aqueous photolysis is another important process organic aerosols undergo in the atmosphere in which their chemical composition and optical properties are altered. This process, including direct photolysis or secondary processes such as hydroxyl radical (OH) oxidation, can transform SOA within cloud or fog droplets during its lifetime in the atmosphere.^19^ Aqueous photolysis can induce photobleaching or photoenhancement through the loss or creation of chromophores, respectively.^19^ Size exclusion chromatography studies have demonstrated that photoenhancement originates from the creation of larger chromophore species during aqueous photolysis.^20^ Given that HULIS is water-soluble, aqueous photolysis is a potentially important process for the transformation of HULIS material in the atmosphere.

In this study, we investigate the photolytic mass loss of SRHA and SRFA as surrogates for ambient SOA material. We use a quartz crystal microbalance (QCM) to monitor the mass change of these materials under 1 day of UV light exposure at three distinct wavelengths: 254, 300, and 405 nm. We focus on the one-day photolysis time frame as previous studies have demonstrated that a majority of the photolytic mass loss of SOA material occurs within the first day in the atmosphere.^1^ We also measured changes in the absorption spectra due to the aqueous photolysis of SRFA using UV–vis spectroscopy. Ultrahigh-resolution mass spectrometry was used to investigate differences in the chemical transformation of SRFA in condensed-phase and aqueous photolysis.

## 2 Material and Methods

In this work, two types of experiments were performed: UV/blue photolysis of SRHA/SRFA on a QCM crystal, and both aqueous and condensed-phase photolysis of SRFA in a photoreactor equipped with UV lamps. The QCM experiments allowed us to measure the photolysis mass loss rate of SRHA/SRFA, while the photoreactor experiments allowed us to capture the evolution of the chemical composition and the absorption spectra of SRFA material during the photolytic aging process.

There are three major differences between SRHA and SRFA: (1) Acidity—SRHA is only fully soluble at pH < 1, whereas SRFA is soluble at all pH.^13^ (2) Molecular Weight—SRHA has a significantly larger average molecular weight compared to SRFA (∼400 Da)^21^ by a factor of around 5.^22^ (3) Aromatic Content—SRHA has a higher aromatic carbon content (31–37%) compared to SRFA (22–24%).^13^ It was concluded that SRFA is the better surrogate for atmospheric HULIS^13^ due to its smaller molecular weight (average ∼300 Da for ambient HULIS),^23^ similar acidic content levels determined by titration,^24^ and comparable aromatic C abundance (19% for ambient HULIS).^13^ In this work, we primarily focus on SRFA because it is a better surrogate for atmospheric application. The condensed-phase photolysis experiments of SRHA were performed to supplement the SRFA experiments. We note that no unexpected or unusually high safety hazards were encountered in carrying out any of the experiments.

### 2.1 QCM Photolysis Experiment

The Suwannee River Humic Acid II (SRHA) and Suwannee River Fulvic Acid I (SRFA) samples were obtained from the International Humic Substances Society (IHSS). These samples were dissolved in 18.2 MΩ Milli-Q water (Millipore Sigma) to form 0.4–0.5 mg/mL aqueous solutions. No adjustment of the pH of the solution was made to attempt to enhance solubility. These solutions were then deposited onto a 2.54 cm diameter chrome/gold quartz crystal (Stanford Research System O100RX1) and allowed to evaporate at 55 °C in an oven overnight. This temperature and concentration combination was determined to ensure that a consistent film of humic substance forms in the active area in the center of the crystal (see Figure S1 in the Supporting Information for more details).

The quartz crystal microbalance (QCM) is a microbalance capable of achieving nanogram-level sensitivity by utilizing the piezoelectric effect of a quartz crystal. In a QCM, a thin quartz crystal disc oscillates at a precise frequency (5 MHz for our instrument) when an AC voltage is applied to it. When mass is deposited onto the active area of the crystal (0.4 cm^2^), its oscillation frequency decreases linearly with the added mass. The masses of the humic substances loaded onto the active area of the crystal were calculated from the measured decrease in QCM oscillation frequency relative to its baseline value. In practice, the humic substance loading on the active area ranged from 4 to 50 μg. The baseline frequency of each crystal was recorded before material was deposited in each run.

**Figure 1:**
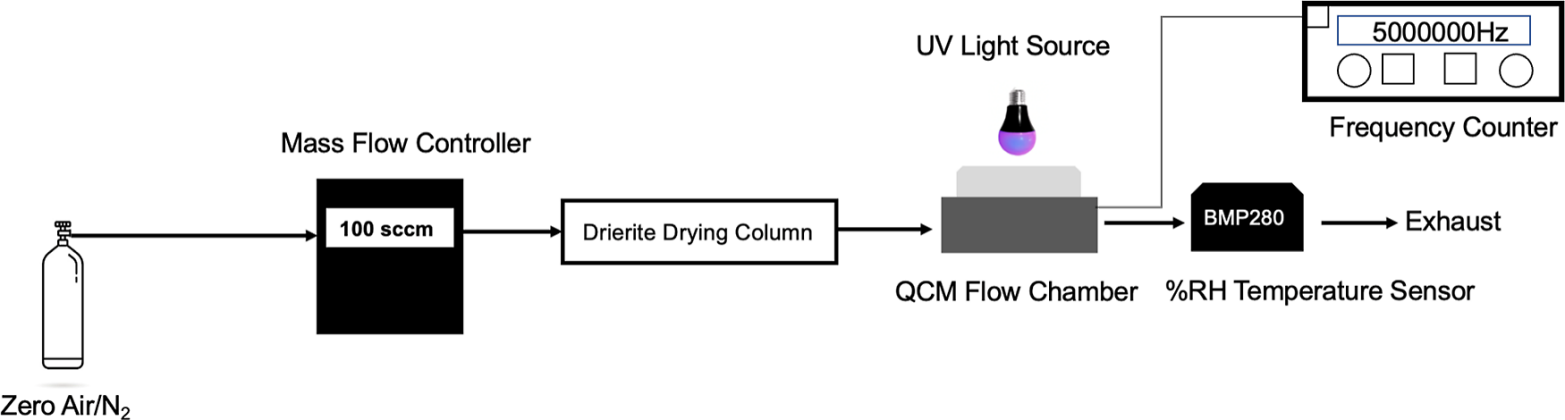
QCM photolysis experiment setup. QCM crystals loaded with humic substances were placed into a crystal holder connecting to the QCM flow chamber adapter. The QCM crystal oscillation frequency was monitored with a frequency counter. The 254 nm mercury pen lamp, 300 nm LED or 405 nm laser was mounted above the flow chamber window to allow irradiation of the sample.

The QCM photolysis flow chamber setup used in this work is illustrated in Figure 1. A commercial QCM holder (Stanford Research Systems QCM100) was connected to a custom stainless-steel adapter with a quartz window on top to allow UV radiation to pass through. This QCM adapter design was inspired by the cell described in Malecha et al.^10^ A purge flow of 100 sccm (standard cubic centimeters per minute) of dry nitrogen or zero air was dried by passing through a Drierite drying column and then sent through the QCM flow chamber. The relative humidity (RH %) in the chamber was maintained below 6% RH as monitored by a sensor (Bosch BMP280) placed inside the outlet tubing.

Three different light sources were selected to span the UV spectrum: a 254 nm mercury pen lamp (UVP 90-0012-01), a 300 nm LED (light emitting diode, Thorlabs M300L4), and a 405 nm diode laser (Coherent 405 LX). For the 254 nm mercury pen lamp, a 250–300 nm bandpass filter (Semrock Brightline 300/SP) was used to eliminate emission lines at wavelengths outside of this range. For the 405 nm laser, the laser beam was expanded with a plano-convex lens to allow full coverage of the crystal active area. The incident power of each light source at the QCM crystal was measured using a power meter (Newport 918D-ST-UV) to be 2.0 mW/cm^2^ for the 254 nm lamp, 2.35 mW/cm^2^ for the 300 nm LED, and 80 mW/cm^2^ for the 405 nm laser, corresponding to fluxes of 2.56 × 10^15^, 3.55 × 10^15^ and 1.63 × 10^17^ photons cm^–2^ s^–1^, respectively.

In the photolysis experiments, the exposure of materials deposited on the crystal to UV radiation resulted in an increase of oscillation frequency, which indicated a decrease in mass of the material. To convert the frequency change to mass change, a simplified version of the Sauerbrey equation was used^25^
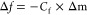
1where Δ*f* and Δ*m* are the changes in oscillation frequency and mass per area in the active area, respectively. *C*_f_ is the sensitivity factor of the QCM crystal, which is 56.6 Hz μg^−1^ cm^2^ for the 5 MHz crystal used in our instrument. This *C*_f_ value was obtained from the SRS instrument manual.^26^ This equation assumes that the mass change occurs uniformly across the entire active area of the crystal. Control experiments demonstrate negligible drift (no more than 0.6%) of the frequency over the course of 24 h for QCM crystals loaded with SRHA or SRFA (Figure S2).

### 2.2 Aqueous Photolysis Experiment

Photolysis experiments were carried out on aqueous samples in a 3.5 mL quartz cuvette (CV10Q3500F, Thorlabs) inside a photoreactor (LZC photoreactor, Luzchem Research) using a 0.13 mg/mL SRFA aqueous solution. The SRFA solution was filtered using a 13 mm PTFE disposable syringe filter (0.2 μm, Omicron Scientific) to remove suspended insoluble materials before use. The photoreactor was equipped with 16 UV lamps (RPR-3000A, S. N. E. Ultraviolet Corp). Spectral fluxes within the photoreactor were characterized with a spectroradiometer (RPS-900, International Light Technologies) and chemical actinometry.^27^ The azoxybenzene actinometry measurements were performed in a cuvette in the same geometry as the aqueous photolysis experiments and used the protocol from Lignell et al. (2013).^28^ Overall, the output of the lamp was determined to range from 290 to 340 nm with a total photon flux of 3.72 × 10^15^ photons/cm^2^/s, which is equivalent to 2.7 times the 24 h averaged photon flux (290–340 nm) in Athens, GA during the summer solstice (6/21/2023). Details about the spectroradiometer measurement, azoxybenzene actinometry and ambient scaling are shown in Figures S3–S5.

Light absorption spectra during photolysis were measured on a double beam UV–vis Spectrometer (Agilent, Cary 60) from 300 to 700 nm at 1 nm resolution. During the 3 h photolysis process, the UV–vis spectrum of the solution was measured every 15 min. In addition, 50 μL of the solution was removed at 0 min, 60 and 180 min for ultrahigh resolution mass spectrometric analysis.

### 2.3 Condensed-phase SRFA Photolysis Experiment

Condensed-phase photolysis was carried out on a SRFA sample that was placed in a beaker in the photoreactor for a duration of 12 h. The sample was prepared by depositing 1 mL of SRFA solution (2 mg/mL, 1:1 water: methanol) in a glass beaker and then drying in an oven at 70 °C for approximately 6 h. After exposure in the photoreactor, the SRFA sample was redissolved in 1 mL of water using sonication for 5 min and then analyzed with ultrahigh-resolution mass spectrometry.

### 2.4 ESI(−)-UHR-MS Analysis

Offline negative ion electrospray ionization ultrahigh-resolution mass spectrometry (ESI(−)-UHR-MS) analysis was performed on a Bruker SolariX 12T FT-ICR to investigate chemical composition change during condensed-phase photolysis of SRFA. Mass spectra were collected over the 100–1500 *m*/*z* range. The transient length was 0.5592 s, which yielded a resolution of 150,000 at 400 *m*/*z*. External mass calibration was performed using sodium trifluoroacetate (NaTFA). Spectra of each sample were acquired at 48 scans averaged per spectra. Peak assignment for the resulting mass spectra was performed using the open-source R package MFassignR.^29^ For each mass spectrum, sample noise was removed using the default KMDNoise function estimation with a signal-to-noise cut off of ≥3. Assigned peak lists were extracted following MFAssignR’s isotope filtering and internal mass calibration steps. All assignments were made with elemental constraints of O ≤ 40, *N* ≤ 3, S ≤ 1, a mass error tolerance of <1 ppm, *m*/*z* within 100–800 range, and limited to singly charged species. After formula assignment, the aromaticity index (AI) was calculated for all formulas^30^
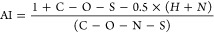
2where C, H, O and S represent the number of corresponding carbon, hydrogen, oxygen and sulfur atoms, respectively, in each formula assignment. The AI value represents the density of double bonds normalized to number of carbons and considers the possible contributions by heteroatoms.^30^ Higher AI values indicate a larger degree of unsaturation, and formulas with AI > 0.5 are considered to represent aromatic compounds.^30^

## 3 Results and Discussion

### 3.1 QCM Photolysis Experiment

**Figure 2:**
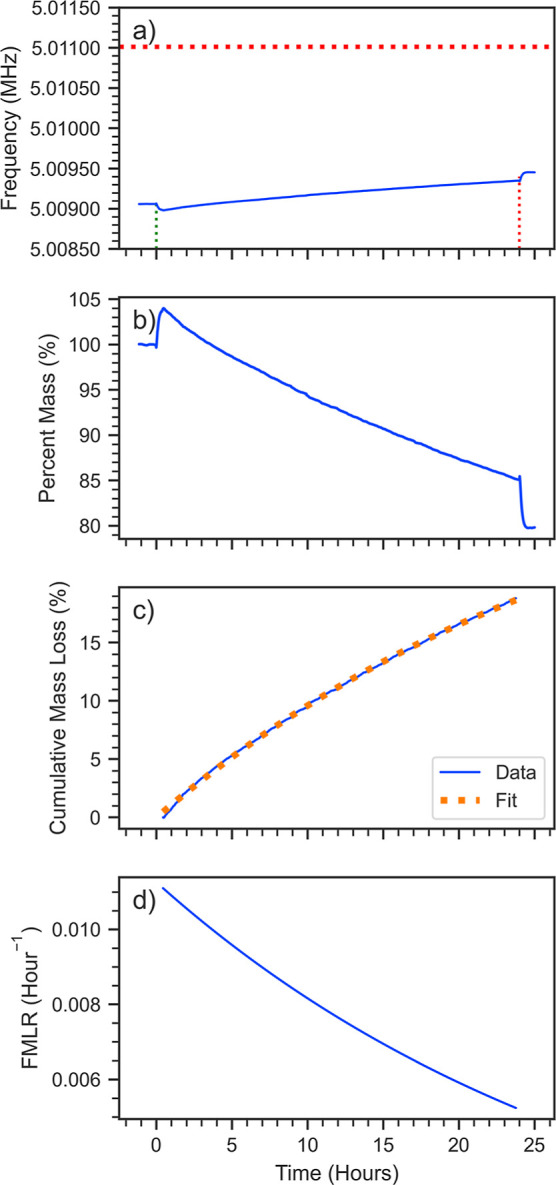
Example of a QCM trace for SRHA exposed to 300 nm radiation. (a) The raw frequency change of an SRHA-loaded crystal (blue curve). Green/red vertical dashed lines indicate the start/end (the 0 and 24 h marks) of the UV exposure. Red horizontal line represents the blank crystal frequency. (b) The percentage mass remaining on the crystal. (c) Cumulative mass loss (CML) percentage during the 24 h exposure with fit to an exponential function including a constant offset: CML = 34.9%–34.5%·*e*^–*t*/31.1h^ (*R*^2^ = 0.9995). (d) Fractional mass loss rate (FMLR) calculated as the derivative of the fit in (c).

Figure 2 shows the results from a typical QCM photolysis experiment in which the frequency of the QCM crystal is monitored (Figure 2a) as the humic substance material is exposed to UV irradiation (300 nm). The baseline frequency of the crystal before mass loading (5.011 MHz) is represented by the horizontal red dashed line. The blue curve represents the frequency of the loaded crystal. Initially, the frequency is seen to decrease when the sample is exposed to the UV radiation but does not correspond to an increase in mass; instead, this is an artifact associated with the heating of the crystal by the 300 nm LED and, in fact, was observed with the 254 nm lamp as well. We confirmed this artifact in control experiments with a blank crystal exposed to the UV radiation as well as to heat from a heat gun (see Figures S6 and S7 in the Supporting Information). For the 405 nm laser, the opposite response was observed with a fast frequency increase accompanying the onset of the exposure to the radiation. We attribute this behavior to the reversible desorption of water on the QCM crystal, which Kawasaki et al. have also observed when exposing a clean QCM crystal to laser irradiation of various wavelengths.^31^ Details of a control experiment that we conducted with a bare QCM crystal exposed to 405 nm radiation are shown in Figure S6. In all cases, the observed artifact does not contribute to photolytic mass loss of humic material, so this reversible artifact was accounted for when calculating the photolysis mass loss rate.

To calculate the mass loss rate due to photolysis, we first converted measured frequency changes to a percent mass remaining (Figure 2b). Then, we calculated the cumulative percent mass loss taking care to account for the heating artifacts (Figure 2c). This curve was then fit to an exponential function with a constant offset (red dashed line) to the data, as used by O’Brien et al. in their study of the photolytic loss of α-pinene SOA.^11^ Finally, the fractional mass loss rate (FMLR; Figure 2d) was calculated as the derivative of this exponential fit. This approach is superior to simply taking the numerical derivative of the raw frequency data to retrieve the photolysis rate, as that approach results in noisy rates that are difficult to interpret. The FMLR figure shown here does not include the initial exposure to the UV radiation because of the heating artifact mentioned previously.

**Figure 3:**
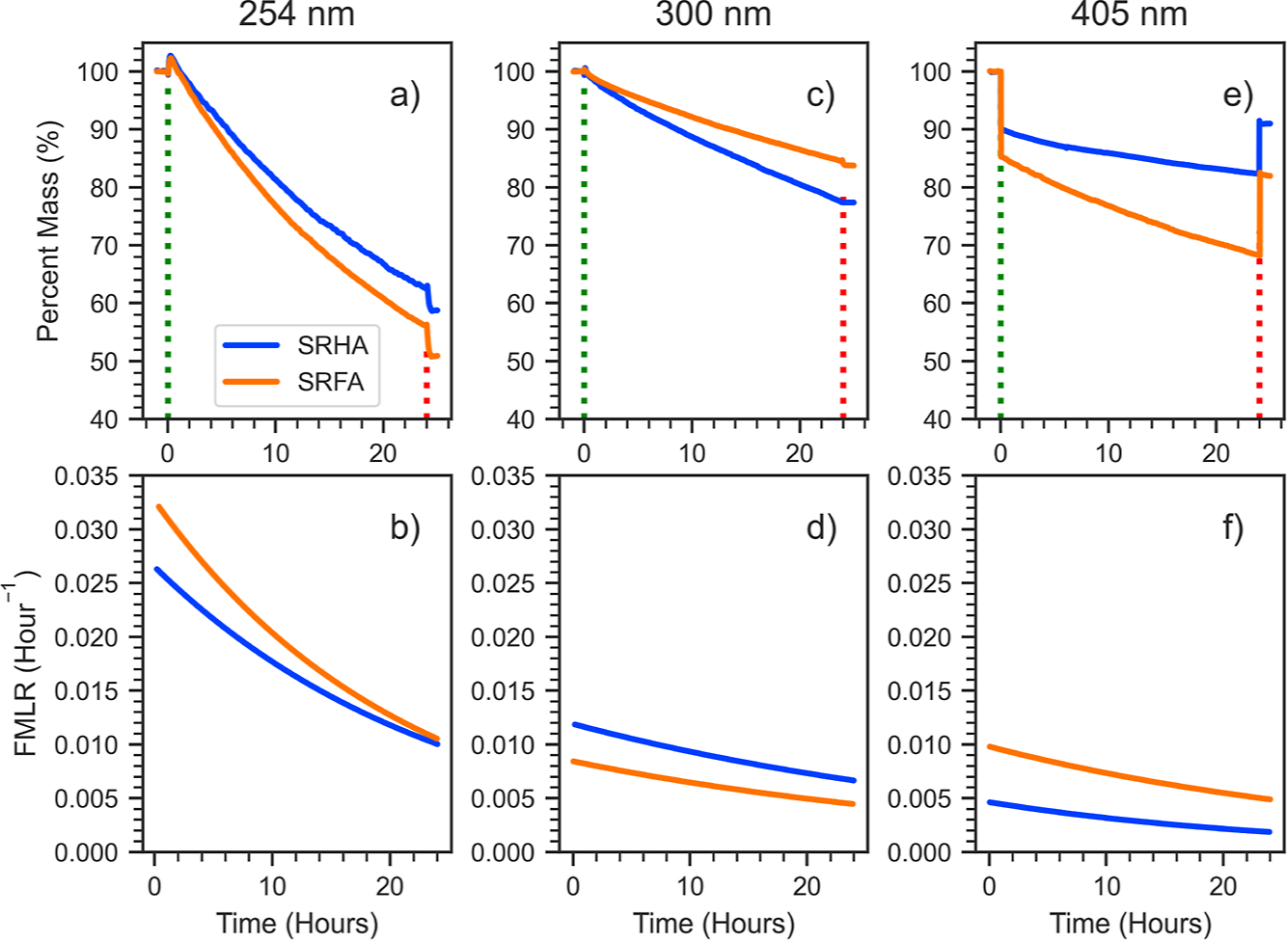
Summary of mass loss experiments performed in zero air. (a,c,e) Percentage mass remaining of SRHA (blue) and SRFA (orange) upon exposure to 254/300/405 nm light, respectively. (b,d,f) Fractional Mass Loss Rate (FMLR) of SRHA and SRFA at the same wavelengths.

Photolysis experiments were conducted at all three wavelengths for both SRHA and SRFA in either zero air or nitrogen. Experiments were repeated three to eight times, and details of all QCM experiment runs are tabulated in Table S1 in the Supporting Information. Figure 3 shows examples of photolysis experiments conducted in zero air. For all three wavelengths, both SRHA and SRFA exhibited a similar trend in photolysis mass loss rate, characterized by a high initial rate that slowly decreased during the 24 h experiment. The time constant, τ, of this exponential decay ranged from 16 to 61 h. Due to this exponential decay shape, naively extrapolating the rate obtained from the first 24 h of exposure linearly to longer times would lead to an overestimation of the mass loss. To illustrate this point, we extended the photolysis experiment of SRFA at 254 and 300 nm to 1 week of light exposure (see Figure S8). At 254 nm, SRFA lost 68% of its mass over the entire week, with 35% lost on the first day alone, while at 300 nm, the loss was 59% over the week compared to 17% on the initial day. Extrapolating the first-day photolysis mass linearly to a whole week overestimated mass loss extent by 41% (254 nm) and 32% (300 nm). Furthermore, the one-week experiment showed that a substantial amount of SRFA mass (approximately 30% for 254 nm radiation) is not lost, consistent with the finding of O’Brien et al. (2019) that a significant fraction of SOA mass is photorecalcitrant.^11^

### 3.2 Oxygen-Induced Mass Loss Acceleration

**Figure 4:**
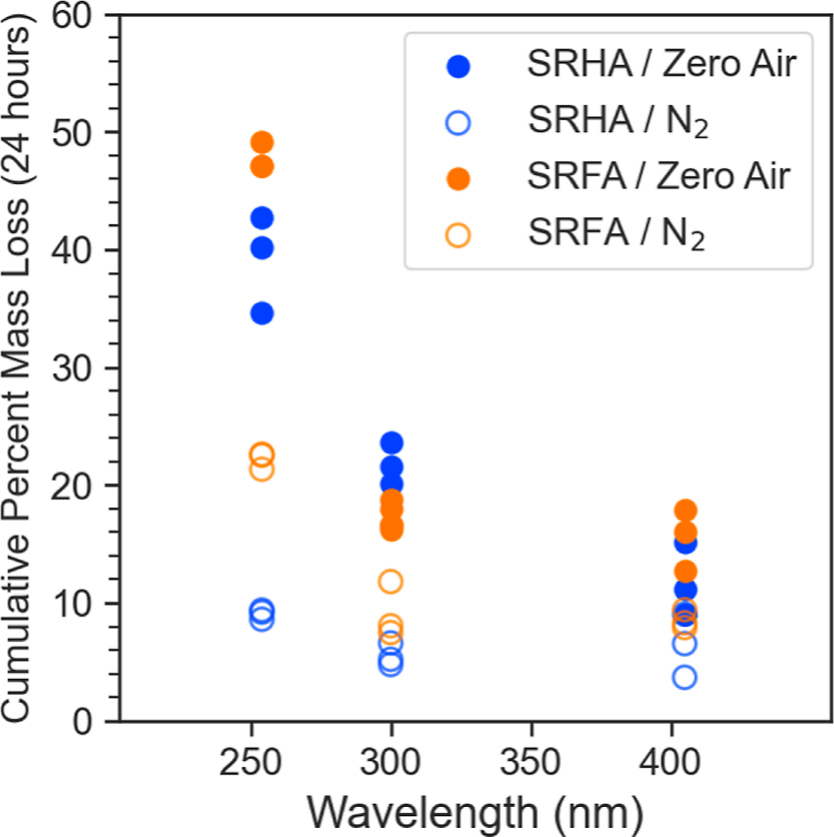
24 h cumulative percent mass loss for SRHA and SRFA in N_2_ and zero air. A substantial increase in mass loss was observed with zero air with all three wavelengths.

We performed the same photolysis experiments in a N_2_ environment to observe the impact of removing O_2_ from the system. The results of these photolysis experiments are summarized in Figure 4, which shows the cumulative mass loss after 24 h of light exposure. A distinct acceleration is evident at all three wavelengths when transitioning from N_2_ to zero air with the magnitude of the effect increasing with decreasing wavelength. For example, at 254 nm, zero air increased mass loss by approximately 2.5× and 4× for SRFA and SRHA, respectively. Given that the primary distinction between N_2_ and zero air is the presence of oxygen, the observed increase is likely attributable to photoinduced oxidation reactions. This result also means that photolysis experiments carried out in N_2_ would lead to an underestimation of the projected mass loss rate in the atmosphere.

One possible explanation for the enhanced rate of mass loss in the presence of O_2_ is the potential for SOA or humic-like material to act as a photosensitizer. Previous studies have demonstrated that natural chromophoric dissolved organic matter (CDOM) possesses photosensitizing properties, enabling it to be promoted to a triplet state (^3^CDOM*) after exposure to solar radiation in the aqueous phase or in the particle.^32^ The ^3^CDOM* generated can subsequently participate in various photochemical processes, such as enhancing secondary organic aerosol (SOA) production through the adsorption of volatile organic compounds (VOCs),^33,34^ promoting reactive oxygen species (ROS) production,^35−37^ reduction of NO_2_ to HONO,^38^ and promoting photoenhancement of biomass burning brown carbon during aqueous and condensed-phase photolysis.^36^ We hypothesize that the photosensitizing capability of humic substances may play a role in the present work as they are known constituents of CDOM.

In a N_2_ environment, mass loss may primarily occur through direct photolysis reactions, such as Norrish type-I or type-II photocleavage of carbonyl structures.^5,10^ In contrast, the presence of oxygen can lead to the formation of ROS produced by photosensitized humic substances, which may enable oxidation of otherwise photostable structures, such as long-chain fatty acids,^39^ and generate more volatile products. In this way, photosensitized ROS formation can lead to an increased number of mass loss pathways and a higher overall mass loss rate. While we are not able to identify specific pathways for the mass loss observed, the higher rates measured in the presence of oxygen indicate that indirect effects, such as humic acid photosensitization, could contribute significantly to SOA mass loss in the atmosphere.

### 3.3 Comparison of Mass Loss Measured for SRHA/SRFA to that of Lab-Generated SOA (from Baboomian et al. (2020))

**Figure 5:**
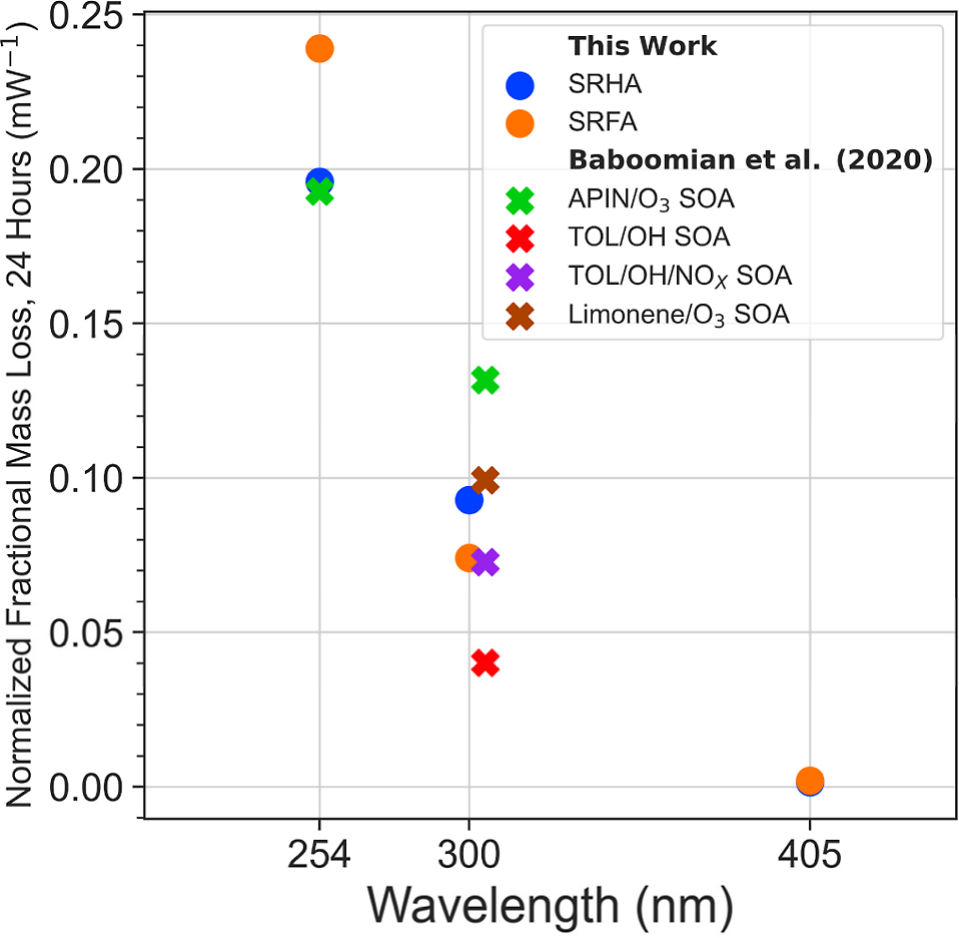
24 h fractional mass loss normalized to light source power in zero air for SRHA and SRFA compared to those from mass loss measurements of lab-generated SOAs from Baboomian et al.^1^

To understand better how the photoinduced mass losses measured for SRHA/SRFA might relate to those of organic aerosols, we compare our results to those of Baboomian et al., who studied lab-generated SOA.^1^ Specifically, they also used a QCM to measure mass loss of particulate matter generated from the oxidation of α-pinene, limonene and toluene precursors upon exposure to 254 and 305 nm light. To compare these experiments, we calculate the 24 h-integrated fractional mass losses in zero air (Figure 5). Here, we normalize the fractional mass loss by dividing the fractional mass loss measured over 24 h by the incident power of the respective light sources to make direct comparisons among wavelengths and between our study and that of Baboomian et al. possible.^1^ It is important to note that the 254 nm measurement in Baboomian et al. did not include the use of an optical filter, as was used in the present work, and therefore may have included contributions from other emission lines from the mercury pen-ray lamp used.^1^ Additionally, we have omitted the 254 nm toluene/OH SOA results reported by Baboomian et al. since they observed complete mass loss in less than 24 h.

In general, we observe a decreasing trend in the normalized mass loss with increasing wavelength indicating that shorter wavelength (higher energy) photons are more effective at inducing mass loss. This trend is most pronounced at 405 nm where, despite measured mass loss of as much as 18% (see Figure 4), the normalized mass losses are negligible. We also find that the normalized mass loss values for SRHA and SRFA are of similar magnitude to those for the lab-generated SOA of Baboomian et al.^1^ Specifically, at 254 nm the values for SRHA and SRFA are within 30% of the value for α-pinene/O_3_ SOA. At 300/305 nm, the values for SRHA and SRFA lie in the middle of the range of values spanned by the biogenic (α-pinene and limonene) and anthropogenic (toluene) SOA. Thus, we conclude that SRHA and SRFA do not preferentially resemble either biogenic or anthropogenic SOA, at least in terms of photolytic mass loss, but they do appear to be reasonable surrogates for SOA, in general, in this respect.

To assess the potential impact that this photolytic mass loss may have on SOA in the atmosphere, we scaled our measurements to the actinic flux during the summer solstice (6/21/2023) in Athens, GA using the approach of Malecha et al.^10^ Details about this scaling process are shown in Figure S9. Utilizing this method, we find that humic/fulvic substances (combining SRHA and SRFA data) would experience a mass loss of 8.25% during the first day in the atmosphere. This rate is equivalent to 0.025% *J*_NO_2__ under the same solar conditions, which is comparable to the value of 0.04% *J*_NO_2__ estimated by Hodzic et al. for SOA.^6,7^ but much smaller than the rates (0.2% *J*_NO_2__ – 2.2% *J*_NO_2__) measured by Zawadowicz et al. for SOA generated from α-pinene and isoprene precursors under dry conditions.^9^ Also, as we demonstrate in Figure 3, the fractional mass loss rates decrease with time, perhaps because of a photorecalcitrant fraction, and as such the 0.025% *J*_NO_2__ value should not be extrapolated to longer time scales.

To put our measured value into context, we compare to the work of Lou et al., who used the Energy Exascale Earth System Model to simulate OA mass concentrations with and without a SOA photolytic sink.^8^ In that work, they demonstrated that a constant SOA photolytic loss rate of just 0.04% *J*_NO_2__ could substantially alter the vertical distribution of the mass concentration of OA and result in better agreement with aircraft measurements in the middle and upper troposphere over the arctic. Likewise, they showed that inclusion of the SOA photolytic loss results in improved agreement of calculated aerosol optical depth of biomass burning aerosols over Africa compared to measurements made with the MODIS satellite. And, in general, they conclude that the magnitude of photolytic loss of SOA is comparable to wet deposition. It is important to note that the contribution by this loss mechanism would be expected to decrease with age in the atmosphere as we observe that the mass loss rate decreases with time. As such, our results suggest that photolytic mass loss might be most important as a sink on short time scales (on the order of a day or two), but that it will not compete with wet deposition over longer time scales of a week or more. Finally, we emphasize that accurate inclusion of this photolytic sink in models would need to account for this dynamic behavior.

### 3.4 Progression of SRFA Absorption Spectra under Aqueous Photolysis

**Figure 6:**
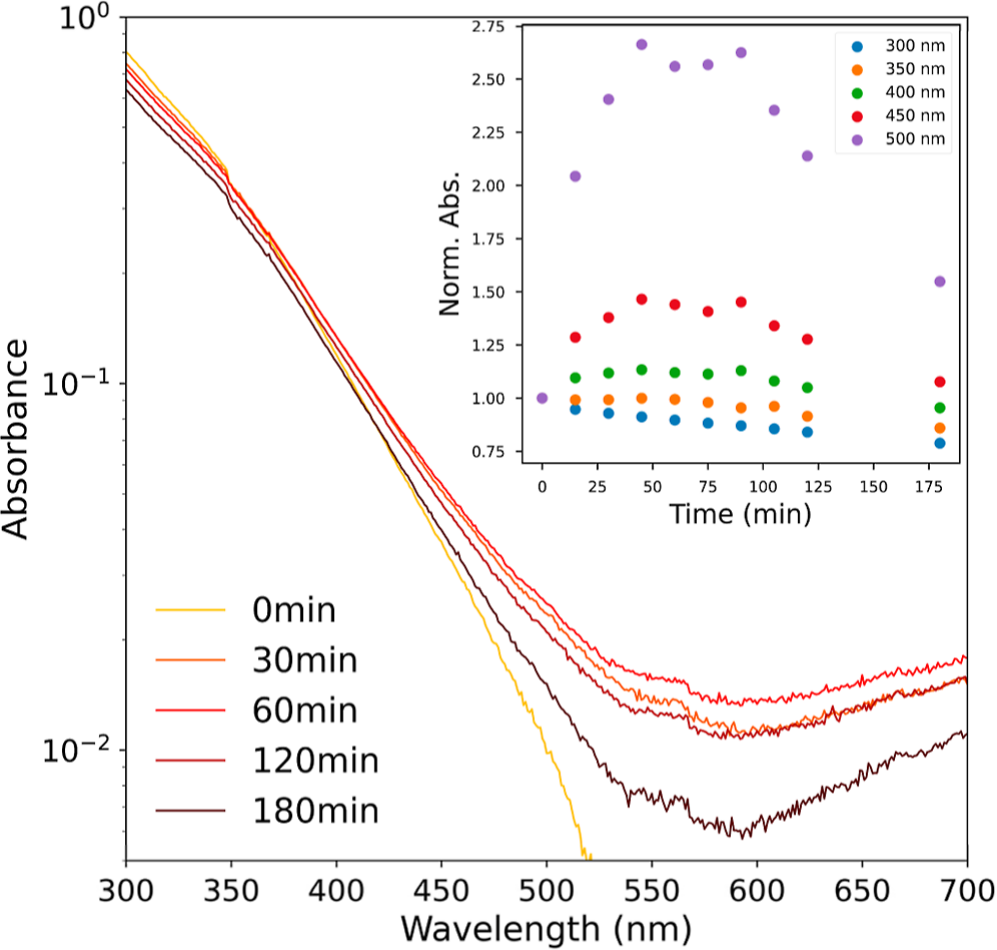
Progression of the aqueous SRFA absorption spectrum with UV photolysis. Photobleaching (at 300–380 nm) and photoenhancement (at 400–700 nm) are both evident. The absorbance scale is limited to values greater than 0.005, the noise level of the UV–vis spectrometer. The inset plots shows the progression of absorption (normalized to 0 min value) observed at 300, 350, 400, 450 and 500 nm.

We characterized the impacts of photolysis on the aqueous SRFA absorption spectrum by exposing the SRFA solution to UV light in a photoreactor. During the photolysis process, both photobleaching in the UV range (300–380 nm) and photoenhancement in the visible range (400–700 nm) was observed (Figure 6). The trajectories of these two effects are different as the photoenhancement effect plateaued at 45–90 min, but the photobleaching effect persisted throughout the entire 3 h of exposure. Overall, the aqueous photolysis of SRFA results in a flattening of the UV–vis spectrum with photobleaching being the dominant effect.

Several studies have examined the effect that light exposure has on the absorption spectra of particulate matter representing various types of organic aerosols. For example, both photobleaching and photoenhancement have been reported for water-soluble brown carbon from wood smoke,^20,36,40,41^ ambient biomass burning brown carbon,^20^ brown carbon from burning of urban construction material,^42^ and 4-nitrophenols^43^ during aqueous photolysis experiments. While the extent to which photolysis modifies these spectra varies, some of them demonstrate the same general pattern that we have observed with SRFA, namely photobleaching at shorter UV wavelengths and photoenhancement at longer visible wavelengths.^36,41−43^ Thus, in this respect SRFA appears to be a good surrogate for organic aerosols.

### 3.5 Evolution of Chemical Composition of SRFA during the Photolysis Process

**Figure 7:**
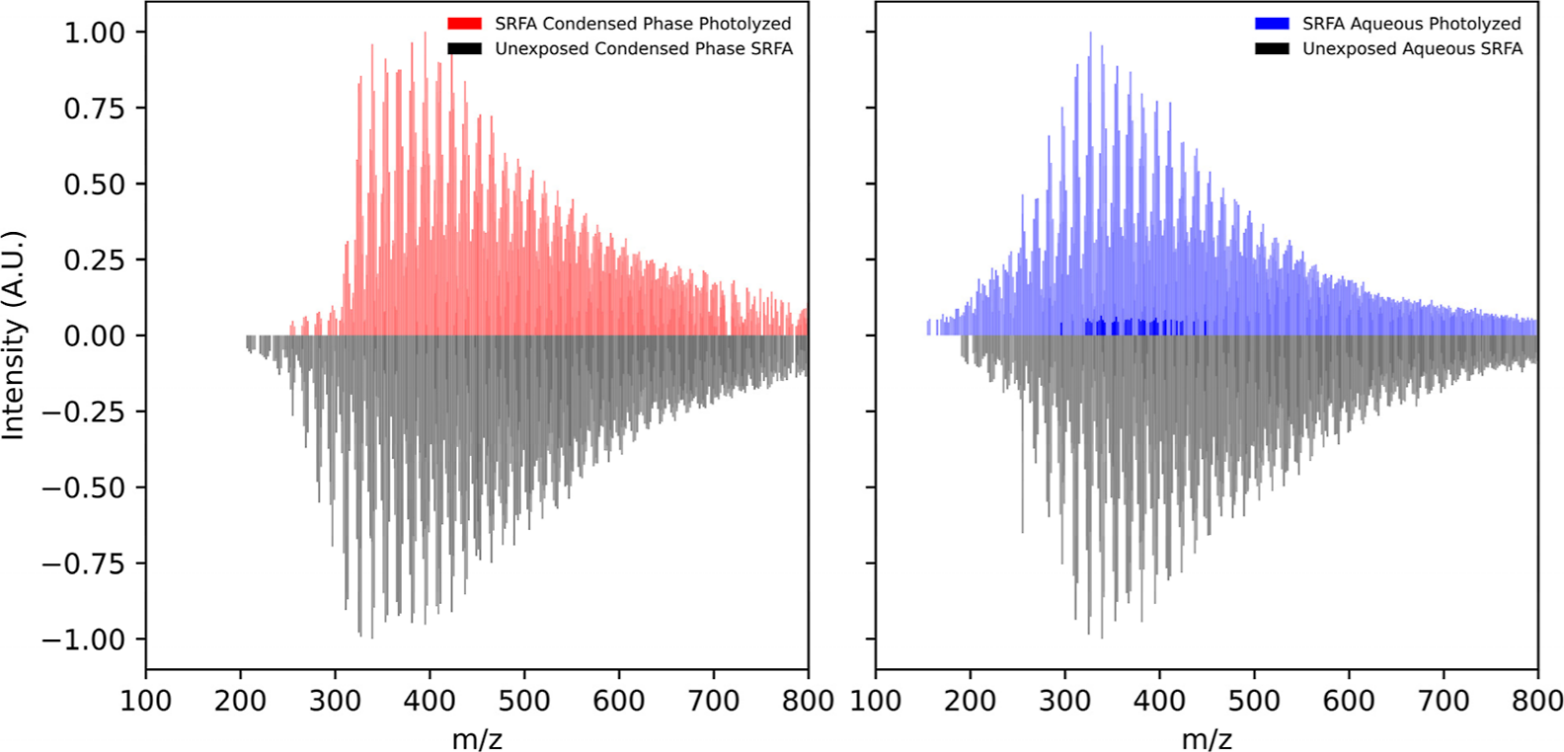
Comparison of the ESI(−)-UHR-MS spectra showing the impact of UV photolysis on condensed-phase (left) and aqueous phase (right) SRFA samples. The mass spectra of the samples prior to exposure to the UV light are displayed as negative-going (black) peaks.

Ultra-High-Resolution Electrospray Ionization Negative Mode Mass Spectrometry (UHR-ESI(−)-MS) was employed to investigate how the chemical composition of SRFA changes under condensed-phase photolysis and aqueous photolysis. Figure 7 displays the mass spectra before and after UV light exposure for both cases. The most noticeable differences between these two cases are found in the 100–300 *m*/*z* range. For the condensed-phase photolysis, the photolyzed sample (red peaks) show a marked decrease in signal intensity in this region, while for aqueous photolysis both the signal intensities and the number of peaks within this range increase (blue peaks). A similar result was observed in the (negative ion) laser desorption ionization (LDI) mass spectra of the condensed-phase SRFA sample with intensity in the 100–300 *m*/*z* range decreasing upon exposure to light (Figure S10) despite the method of ionization being different.

In general, molecules with smaller *m*/*z* values tend to be more volatile,^44^ and the differences observed for the condensed-phase and aqueous samples suggest different fates for these higher-volatility species. In the aqueous phase, the increase in intensity of these low-*m*/*z* peaks implies that they are created as a result of the photolysis and then retained in the solution. On the contrary, for the condensed-phase sample the marked decrease in intensity suggests that any such low-*m*/*z* products that are generated evaporate. That decrease also implies that low-*m*/*z* species that were present before exposure to light participated in photoinitiated reactions, either leading to products with higher volatility that subsequently evaporated or to products with larger *m*/*z* values. While it is not possible to identify conclusively from the mass spectra which of these possibilities is more likely, it appears that the larger *m*/*z* region (*m*/*z* > 300) is largely the same before and after exposure to light suggesting that the loss of low-*m*/*z* intensity is a result of product evaporation.

The differences observed in the condensed-phase and aqueous samples could have implications for organic particulate matter in aqueous droplets in the atmosphere. The low-*m*/*z* products created from photolysis within droplets could evaporate when the droplets subsequently evaporate, thereby constituting a delayed loss of mass from the particulate matter. Additionally, these low-*m*/*z* products may also be more likely to be involved in photoinitiated reactions, as evidenced by the notable loss in intensity in the condensed-phase mass spectrum (Figure 7a), in which case the aqueous phase photolysis could essentially be “priming” the condensed-phase photolysis. Future work could investigate this hypothesis by subsequently exposing previously photolyzed aqueous samples after evaporating the water from them.^46^

As a way to interpret the changes in the mass spectra accompanying exposure to light, we calculate the aromaticity index (AI; eq 2),^45^ which is a measure of the “density” of carbon–carbon double bonds within a molecule.^45^ Compounds with higher values of the aromaticity index are more likely to have greater extent of conjugation, which could indicate that they are more likely to act as chromophores,^46^ and AI values greater than 0.5 indicate the presence of aromatic structures.^45^

**Figure 8:**
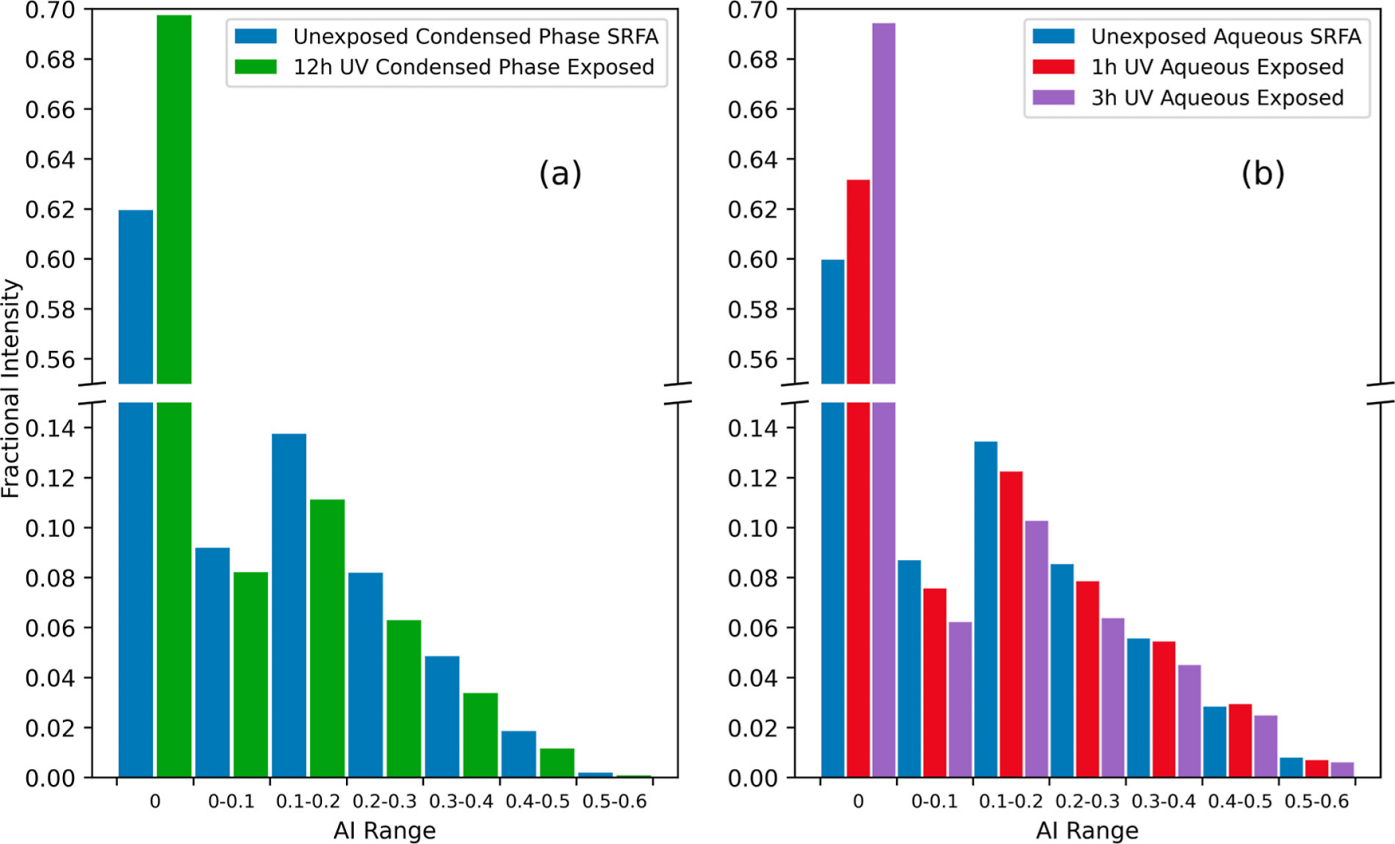
Distribution of aromaticity index (AI) of SRFA before and after: (a) UV condensed-phase photolysis and (b) UV aqueous phase photolysis. The fractional intensity in each bin is calculated as the sum of the intensities for all peaks in the bin normalized to the sum of the intensities for all peaks in the mass spectrum.

In Figure 8, we show the distribution of AI values calculated for formulas identified in mass spectra before and after exposure to UV radiation. For both the condensed-phase and aqueous samples, we observe that before photolysis most of the identified formulas correspond to AI = 0. After photolysis, the fraction of formulas with AI = 0 is even greater with a concomitant decrease in the fractions with AI > 0, including the AI > 0.5 fraction that indicates true aromaticity. These changes are consistent with a decrease in the extent of conjugation present and the UV photobleaching observed in the UV–vis spectra (Figure 6).

The observed decrease in the values of the AI is the opposite of what Baboomian et al. found for lab-generated SOA material using ESI(+)-HR-MS analysis in which they observed an increase in abundance of larger aromaticity values.^1^ The discrepancy between these two studies may be attributable to the selectivity difference in the two different ionization modes. For example, it has been demonstrated that ESI(+) favors aliphatic structures (AI = 0), while ESI(−) can detect more formulas with conjugated structures (AI > 0).^46^ This difference highlights the potential bias and limitation of relying on only one ionization mode in mass spectrometric analysis of complex mixtures since the peaks detected and their intensities measured are functions of both the ionization technique used and any sample matrix effects.^47^ Moving forward, it would be advantageous to utilize combined information from different ionization modes making more comprehensive conclusions possible.

## 4 Conclusions

Four implications follow from the present work:

(1) The mass loss rates observed upon exposure to 300 nm light for SRHA and SRFA are comparable to those of laboratory-generated secondary organic aerosol (SOA) from α-pinene, limonene and toluene precursors.^1^ This finding reinforces the validity of using SRHA/SRFA as surrogates for atmospheric SOA and HULIS, at least with respect to photolytic mass loss, which is beneficial as SRHA/SRFA material is readily available to purchase and does not require generation through photo-oxidation in chamber experiments in the lab. The use of SRHA/SRFA also makes it more straightforward to conduct experiments in a reproducible manner. However, one must be cautious as SRHA/SRFA tend to have larger molecular weights than HULIS.^13^
(2) The measured mass loss rate of 0.025% *J*_NO_2__ for SRHA/SRFA for the first day in the atmosphere is comparable to the value of 0.04% *J*_NO_2__ estimated for SOA by Hodzic et al.^6^ Additionally, our measured value confirms the findings of Lou et al., namely that photolytic loss is a significant sink for SOA that may be on par with wet deposition over the time scale of a day or two.^8^ However, the fact that we observed this rate decrease with time also emphasizes the potential dynamic nature of this loss process that must be considered when incorporating into models.
(3) Condensed-phase photolysis is more than twice as fast under a zero air environment compared to a N_2_ environment, which is attributable to the presence of oxygen. These results show that oxidation-related processes are responsible for more than 50% of the mass loss. The observed increase in mass loss may originate from the potential for humic/fulvic material to act as photosensitizers in a manner similar to that observed for chromophoric dissolved organic matter (CDOM) generating ROS under exposure to sunlight.^32^ It should be noted that our current approach does not measure ROS production during photolysis, and the importance of this potential photosensitizer behavior of humic substances is still an open question. In addition, our results suggest that any experiments conducted in a N_2_ environment could yield an underestimation in the rate of photodegradation due to absence of O_2_.
(4) The mass spectra of SRHA/SRFA samples after undergoing aqueous photolysis and condensed-phase photolysis are different. Specifically, aqueous photolysis results in a slight increase in signal intensity in the low *m*/*z* range (100–300 *m*/*z*), whereas condensed-phase photolysis results in reduced intensity in that same range. Species in this range are likely to be more volatile, which would imply that additional mass loss could occur when aqueous droplets containing particulate matter evaporate in the atmosphere. Future studies could investigate these effects by evaporating aqueous solutions after they have been exposed to UV light and also by subsequently exposing the dried samples to UV light.

## Supporting Information Available

Summary of all QCM experiments, details of QCM control experiments investigating light source artifact, photographs of sample deposition on QCM crystal, QCM drift control experiments, results of a one-week SRFA photolysis experiment, scaling of mass loss rate to typical actinic flux, characterization of the photoreactor, LDI(−)-HR-MS results for SRFA (PDF)
