# Supplementary material for: Photolytic Mass Loss of Humic Substances Measured with a Quartz Crystal Microbalance
Source: ACS Earth Space Chem. 2024 Jul 11;8(8):1623–33. doi: 10.1021/acsearthspacechem.4c00134 (PMC11331507; doi:10.1021/acsearthspacechem.4c00134)
Supplement: Supplementary file 1 — sp4c00134_si_001.pdf [file sp4c00134_si_001.pdf]

Supporting Information

Photolytic Mass Loss of Humic Substances Measured with a Quartz Crystal Microbalance

Mingrui Sun and Geoffrey D. Smith*
Department of Chemistry, University of Georgia, Athens, Georgia 30602, USA
* Email: geosmith@uga.edu, Phone: +1 706-583-0478

Table of Contents

Table S1. Summary of all QCM experiments in this work 3

Figure S1. Photographs of Humic Substance Deposition to QCM Crystal 5

Figure S2. Control Experiment for QCM baseline drift 6

Figure S3, S4, S5. Photoreactor Characterization 7

Figure S6, S7. Control Experiment of light source opening/closing and heating artifacts 10

Figure S8. Result of One Week SRFA Photolysis Experiment under 254/300nm radiation 12

Figure S9. Exponential Fit of Photon Flux Normalized Mass Loss Rate and Solar Spectrum Used for Atmospheric Condition Scaling 13

Figure S10. Laser Induced Ionization (LDI) HR-MS of SRFA sample 15

References 16

Table S1. Summary of all QCM experiments in this work

Sample	λ (nm)	Gas	Time	Photon Flux (Photons cm^{-2} s^{-1})	Mass Loaded (μg)	Overall Total Mass Loss (%)	Fitted Time Constant (hour)**	Max FMLR (%/hour)	Min FMLR (%/hour)
SRHA	254	N2	24	2.56E+15	48.0	8.6	10.15±0.1	0.8	0.1
SRHA	254	N2	24	2.56E+15	48.3	8.9	7.0±0.2	0.8	0.0
SRHA	254	N2	24	2.56E+15	58.4	9.4	8.8±0.1	0.9	0.1
SRHA	300	N2	24	3.55E+15	63.4	4.8	14.2±0.2	0.3	0.1
SRHA	300	N2	24	3.55E+15	38.3	6.6	8.4±0.1	0.7	0.0
SRHA	300	N2	24	3.55E+15	66.9	5.2	15.8±0.2	0.4	0.1
SRHA	405	N2	24	1.63E+17	68.4	3.7	9.1±0.1	0.3	0.0
SRHA	405	N2	24	1.63E+17	28.2	6.5	14.4±0.1	0.4	0.1
SRHA	405	N2	24	1.63E+17	25.1	9.0	11.7±0.1	0.7	0.1
SRHA	254	Zero Air	24	2.56E+15	10.6	42.7	24.2±0.1	2.6	1.0
SRHA	254	Zero Air	24	2.56E+15	14.2	34.6	24.5±0.2	2.0	0.8
SRHA	254	Zero Air	24	2.56E+15	12.1	40.2	24.8±0.2	2.6	1.0
SRHA	300	Zero Air	24	3.55E+15	13.8	20.1	31.2±0.2	1.1	0.5
SRHA	300	Zero Air	24	3.55E+15	10.1	23.7	40.3±0.4	1.2	0.7
SRHA	300	Zero Air	24	3.55E+15	11.8	21.6	51.0±0.6	1.0	0.6
SRHA	405	Zero Air	24	1.63E+17	15.3	9.0	26.17±0.3	0.5	0.2
SRHA	405	Zero Air	24	1.63E+17	69.7	11.1	29.2±0.3	0.6	0.3
SRHA	405	Zero Air	24	1.63E+17	4.6	15.2	NA*	NA*	NA*
SRFA	254	N2	24	2.56E+15	19.9	16.7	8.4±0.1	2.2	0.1
SRFA	254	N2	24	2.56E+15	17.3	22.8	9.2±0.1	2.0	0.2
SRFA	254	N2	24	2.56E+15	26.9	22.5	9.2±0.1	2.1	0.2
SRFA	300	N2	24	3.55E+15	14.4	11.8	14.7±0.1	0.9	0.2
SRFA	300	N2	24	3.55E+15	24.8	7.5	16.9±0.2	0.5	0.1
SRFA	300	N2	24	3.55E+15	29.6	8.1	14.7±0.2	0.6	0.1
SRFA	405	N2	24	1.63E+17	21.2	9.4	15.2±0.1	0.6	0.1
SRFA	405	N2	24	1.63E+17	19.2	7.9	21.8±0.2	0.4	0.1
SRFA	405	N2	24	1.63E+17	23.6	8.3	18.1±0.1	0.5	0.1
SRFA	254	Zero Air	24	2.56E+15	13.4	49.2	21.2±0.1	3.2	1.0
SRFA	254	Zero Air	24	2.56E+15	14.6	47.1	22.2±0.1	3.0	1.0
SRFA	254	Zero Air	24	2.56E+15	12.9	47.1	24.2±0.1	3.0	1.1

SRFA	300	Zero Air	24	3.55E+15	29.7	18.8	37.3±0.5	1.0	0.5
SRFA	300	Zero Air	24	3.55E+15	25.4	16.3	37.5±0.3	0.8	0.4
SRFA	300	Zero Air	24	3.55E+15	28.8	16.5	37.5±0.3	0.9	0.5
SRFA	300	Zero Air	24	3.55E+15	31.5	16.5	49.8±0.7	0.8	0.5
SRFA	300	Zero Air	24	3.55E+15	32.6	18.1	37.8±0.6	0.9	0.5
SRFA	300	Zero Air	24	3.55E+15	32.6	16.6	35.5±0.5	0.8	0.4
SRFA	300	Zero Air	24	3.55E+15	20.3	16.6	52.8±0.8	0.8	0.5
SRFA	405	Zero Air	24	1.63E+17	24.7	13.7	25.0±0.2	0.9	0.3
SRFA	405	Zero Air	24	1.63E+17	11.7	16.1	34.5±0.2	1.0	0.5
SRFA	405	Zero Air	24	1.63E+17	17.1	12.4	16.4±0.1	0.8	0.2
SRFA	254	Zero Air	168	2.56E+15	20.7	68.0	33.4±0.02	2.0	0.01
SRFA	300	Zero Air	168	3.55E+15	20.0	59.0	80.3±0.02	0.8	0.1

*: This run experienced network outage during the first hour after light source on, so no fit was performed.

**: Error Bar on the fitted constant was obtained through Python Scipy package curve fit function

Figure S1. Photographs of Humic Substance Deposition to QCM Crystal:

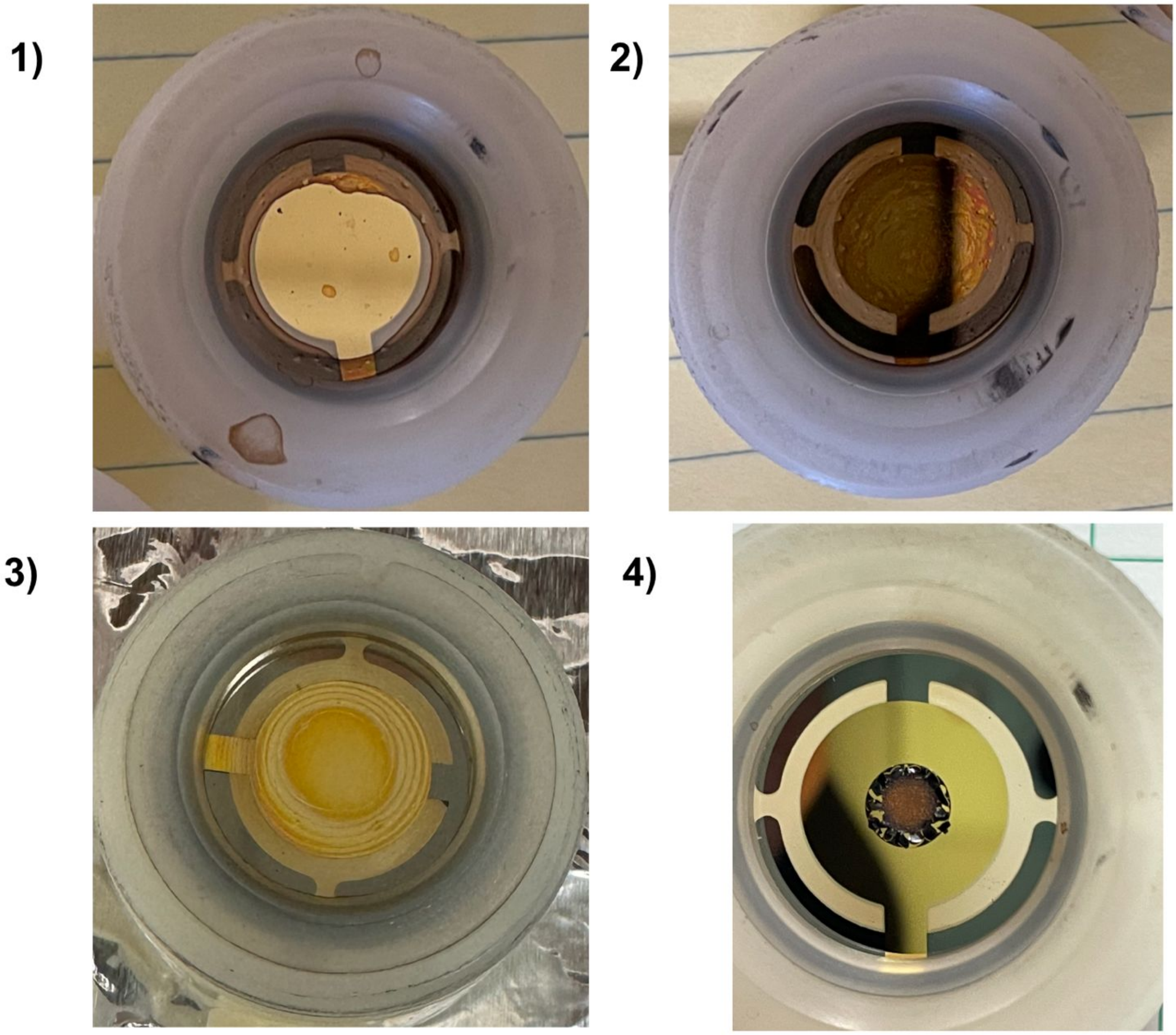

Figure S1: Photographs of humic substance films prepared on QCM Crystal. 1) Inadequate coverage of the active region (center gold-coated area) due to excessively high evaporation temperature (~75°C). 2) Example of a humic substance film prepared with an optimal evaporation temperature of ~55°C and suitable solution concentration. 3) SRHA film deposited on the crystal under appropriate concentration (0.4-0.5 mg/mL) and evaporation temperature (55 °C). 4) Excessively high humic substance solution concentration resulting in a flaky film that did not adhere properly to the crystal. Note that examples 1), 2), and 4) were prepared using Fluka humic acid for testing and demonstration purposes.

Figure S2. Control Experiment for QCM baseline drift

Blank experiments of loading the QCM crystal with humic substances and recording its frequency change without UV light exposure for 24 hours are summarized in Figure S2. The measured frequency drift ranged from 0.676 Hz to 13.649 Hz and varied from run to run. The magnitude of the drift over 24 hours is similar to our instrument stability of less than than 0.1 Hz/hour (12 Hz/day) drift for a clean crystal (SRS instrument manual).[2] This drift is negligible compared to the overall frequency increase due to photolysis (260-1060 Hz frequency increase, or 3.5-49% frequency increase normalized by loading) but varies between experiments. In this instance, we attribute all raw frequency changes to photolysis without considering this drift, as it is insignificant and inconsistent.

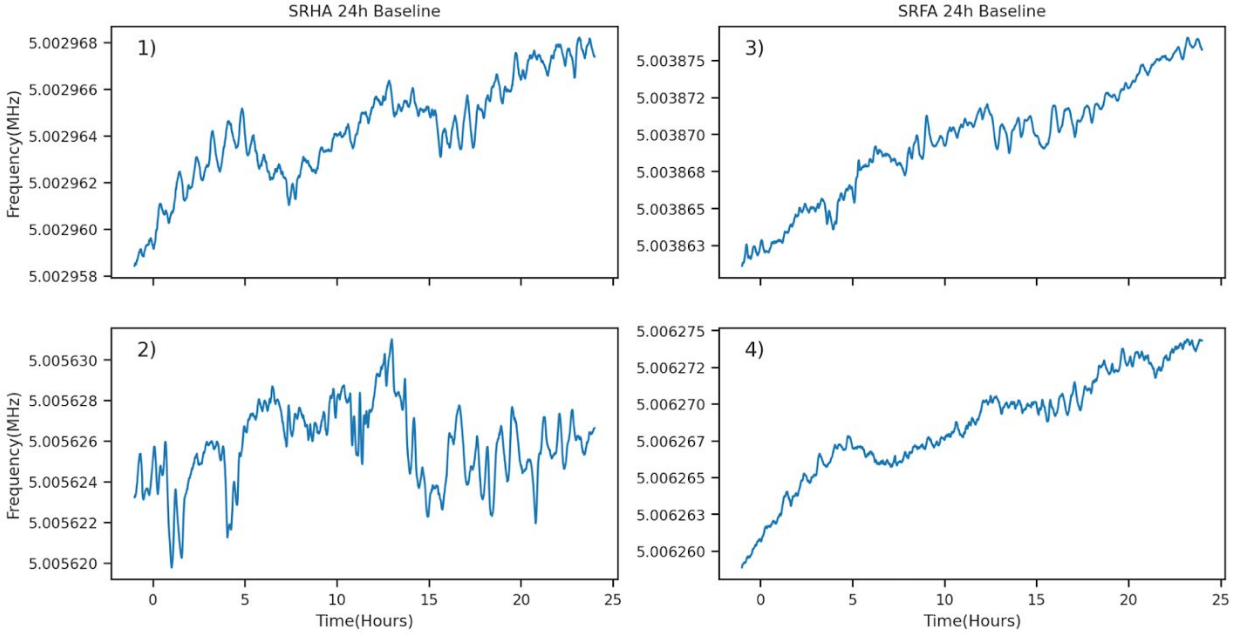

Figure S2. Baseline of SRHA/SRFA loaded crystal during 24-Hour blank experiment. Pairs 1)/2) and 3)/4) represent two separate experiments for SRHA and SRFA, respectively. The material loading on each crystal and the 24-hour frequency drift are as follows: 1) 8.171 Hz drift vs 2579.24 Hz loading (drift ~ 0.3% /day); 2) 0.676 Hz drift vs 1011.355 Hz loading (drift ~ 0.07%/day); 3) 13.649 Hz drift vs 4452.613 Hz loading (drift ~ 0.3%/day); 4) 12.589 Hz drift vs 2053.45 Hz loading (drift ~ 0.6%/day).

Figure S3, S4, S5. Photoreactor Characterization

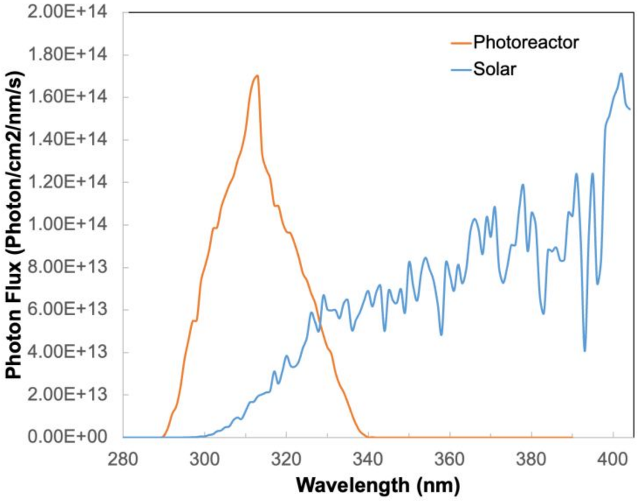

Figure S3. Spectrum of lighting condition within the photoreactor setup compared to Athens, GA's 06/21/2023 24-hour average solar spectrum obtained from "Quick TUV" calculator.[3] The UV lamp spectral shape was obtained with a spectroradiometer (RPS-900, International Light Technologies) and then scaled using the azoxybenzene chemical actinometer measurement.[4] Details of how the spectrum was measured are shown in Figure S4. The ambient scaling factor was determined by taking ratio between the integration of the photoreactor spectrum over the solar spectrum in the 290 – 340 range. This scaling factor is determined as 2.7 in this case.

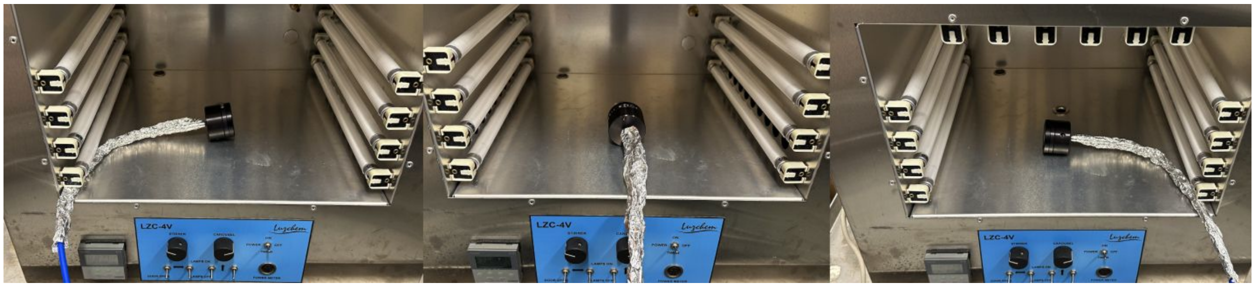

Figure S4. Demonstration of spectroradiometer measurement in the photoreactor. Emission spectrum inside the UV reactor was measured with a spectroradiometer equipped with a cosine diffusor facing to the left, center, right of the chamber. Overall emission spectrum experienced by the cuvette was weighted average of these three measurements ((center*2 + left + right)*0.25). Sensor head pointing up wasn't measured as the cuvette had a plastic stopper which would block direct emission from the top. Finally, the spectral shape obtained was scaled using the chemical actinometer measurement resulting in the scaled spectrum shown in Figure S3.

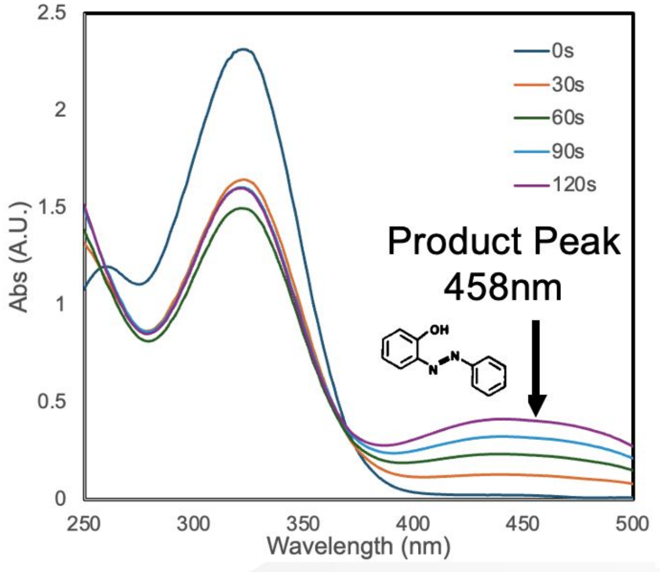

Figure S5. UV-Vis spectra from the azoxybenzene actinometer experiment. A photo-isomerization product peak with a known molar extinction coefficient is highlighted.

The azoxybenzene actinometer experiment was conducted using an azoxybenzene ethanol solution with 0.2 mM azoxybenzene and 0.6 mM KOH. 3 mL of this solution was added to the cuvette placed in the center of the photo-reactor. During 2 min UV exposure, the UV-Vis absorption spectrum of the solution was collected every 10 s. The azoxybenzene photo-isomerization product has a known molar extinction coefficient at 458 nm of 7600 L mol^{-1} cm^{-1},[4] which is used to convert the absorption to product concentration. The photon flux in the UV range is given by:[5]

$$A_0 \ln\left(1 - \frac{P}{A_0}\right) = -\phi_r I_0 t$$

in which A_0 is the initial azoxybenzene concentration, P is the photo-isomerization product concentration, ϕ_r is the quantum yield of product formation for this reaction (a constant 0.21 in the UV range),[5] t is the duration of this reaction and I_0 is the photon flux in units of concentration. This equation is linear up to 40% conversion of the product.[5]

To convert I_0 to F_0, photon flux in units of photon/cm^2/s:

$$F_0 = I_0 * V * \frac{N_A}{Area}$$

in which V is the volume of solution (3 mL), N_A is Avogadro's number and Area is the area of the cuvette being exposed to light. In our case, Area = 12 cm.

Overall, the photon flux inside the chamber was determined to be 3.72 * 10^{15} photons/cm^2/s. This value was then used to scale the spectral shape obtained with the spectroradiometer (Figure S3).

Figure S6, S7. Control Experiment of light source opening/closing and heating artifacts:

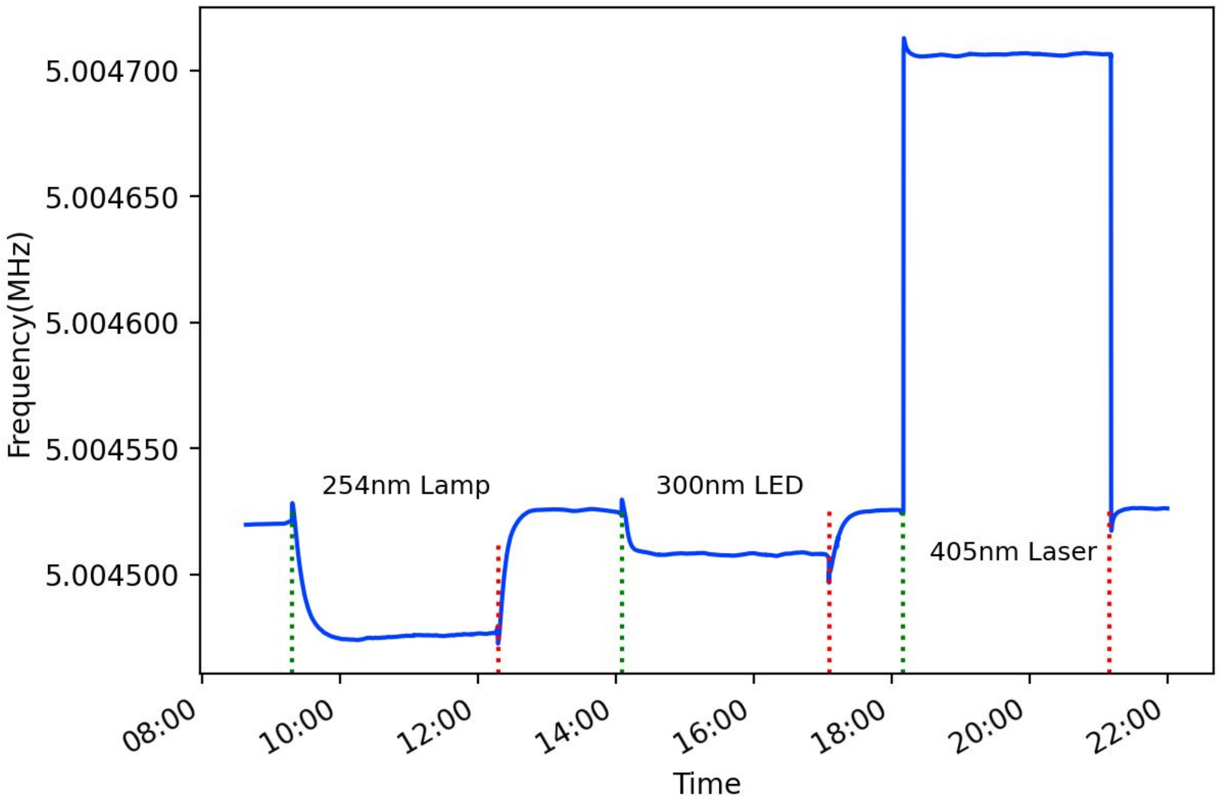

Figure S6: Light On-Off response of a bare crystal. A three-hour On-Off cycle (indicated by green and red dotted lines) was conducted with 254/300/405 nm light sources, allowing time between cycles to ensure the baseline stabilized.

The 254 nm lamp and 300 nm LED produced similar artifacts: frequency initially spiked up and then decreased after the light was turned on, and the reverse changes occurred after the light was turned off. Heat generated by the light source was responsible for this type of artifact, as it was a slow process taking more than 10 minutes to stabilize. The 254 nm lamp produced more heat than the 300 nm LED, resulting in a larger frequency shift. This was also confirmed in a heat gun experiment shown in Figure S7.

The 405 nm On-Off artifact differed significantly from the lamp or LED, displaying a rapid frequency spike up or down when the laser was on or off, which suggests a different mechanism compared to the lamp or LED. Kawasaki et al. (2009) explained this artifact through photo-induced reversible desorption of water

molecules from the crystal's gold surface.[1] Since this process is reversible, it does not contribute to mass changes due to photolysis.

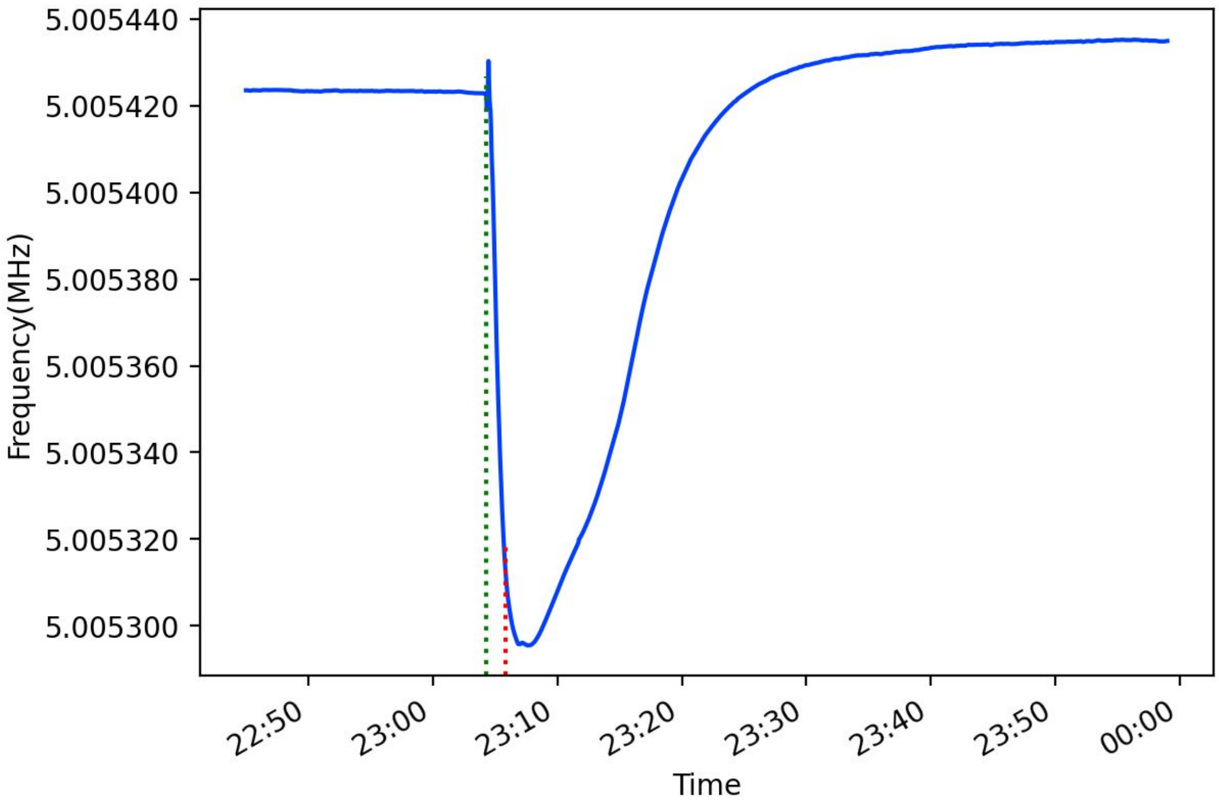

Figure S7: Heat gun control experiment with SRFA-loaded crystal. A 2-minute On-Off cycle (indicated by green and red dashed lines) was performed using a heat gun to blow hot air into the QCM enclosure.

Similar to the light On-Off experiment (Figure S6), the frequency initially spiked, then decreased with heating, and finally recovered to a higher baseline after the heat was turned off. It is important to note that the heat gun employed in this experiment generated more heat than the light source, resulting in larger magnitude artifacts compared to Figure S6. The observed frequency lag when the heat was turned off can be ascribed to the heat transfer process between the enclosure's exterior and the crystal. The elevated baseline (approximately 12 Hz change) following the On-Off cycle is believed to be due to heat-induced evaporation of the SRFA material. Overall, the artifacts produced by the heat gun were consistent with those observed using the 254/300 nm light source, further corroborating heat as the source of that artifact.

Figure S8. Result of One Week SRFA Photolysis Experiment under 254/300nm radiation

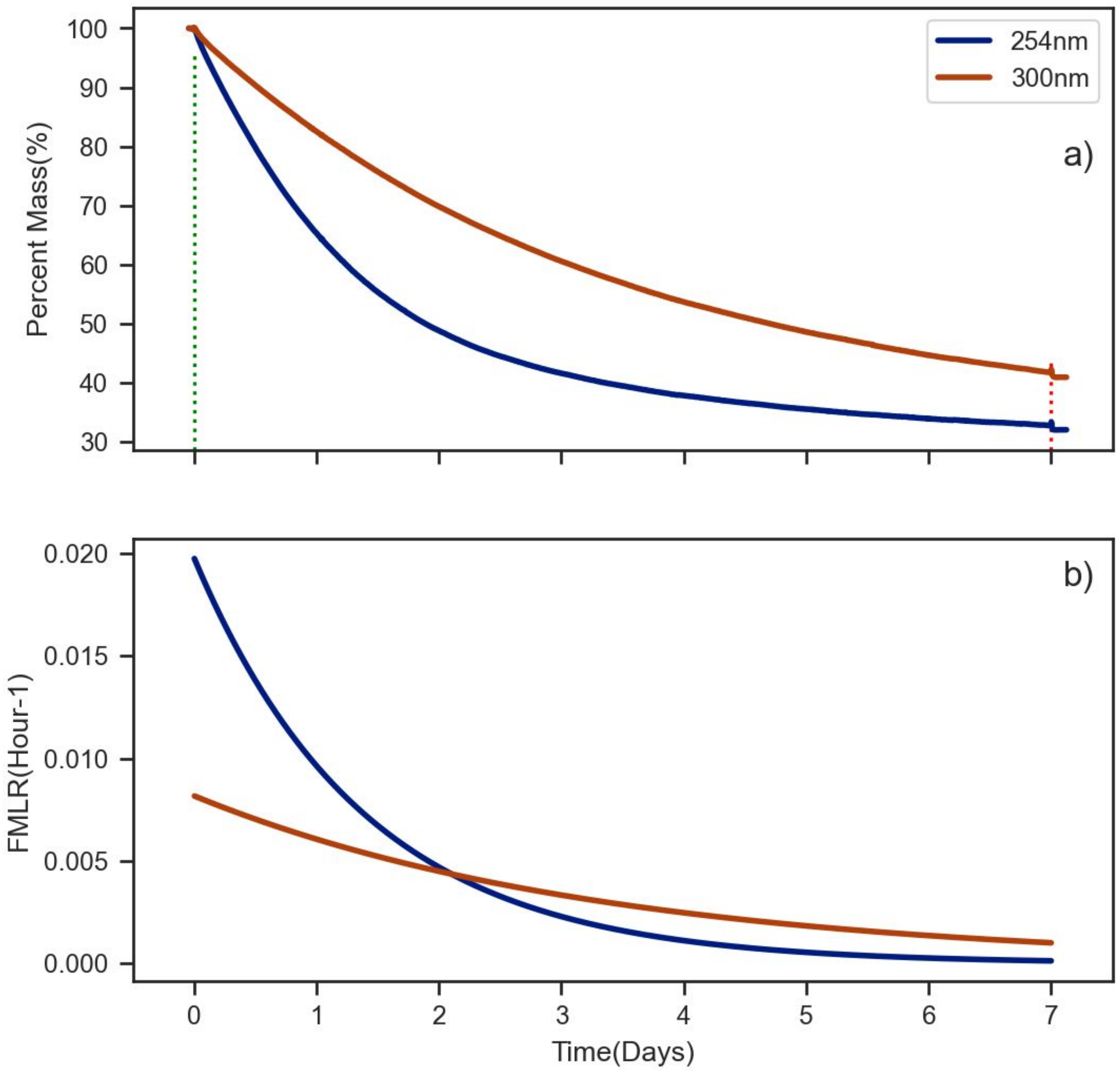

Figure S8. Summary of SRFA one week photolysis mass loss experiment under 254/300nm light exposure. (a) overall percent mass on the crystal and (b) fractional mass loss rate over the week. Overall, the sample exposed to 254 nm light loses 68.0% of its mass, while the sample exposed to 300 nm light loses 59.0% of its mass. In addition, the majority of these losses occurred early on, with 51% and 30% of the overall mass loss for 254 nm and 300 nm light, respectively, occurring in the first day.

Figure S9. Exponential Fit of Photon Flux Normalized Mass Loss Rate and Solar Spectrum Used for Atmospheric Condition Scaling

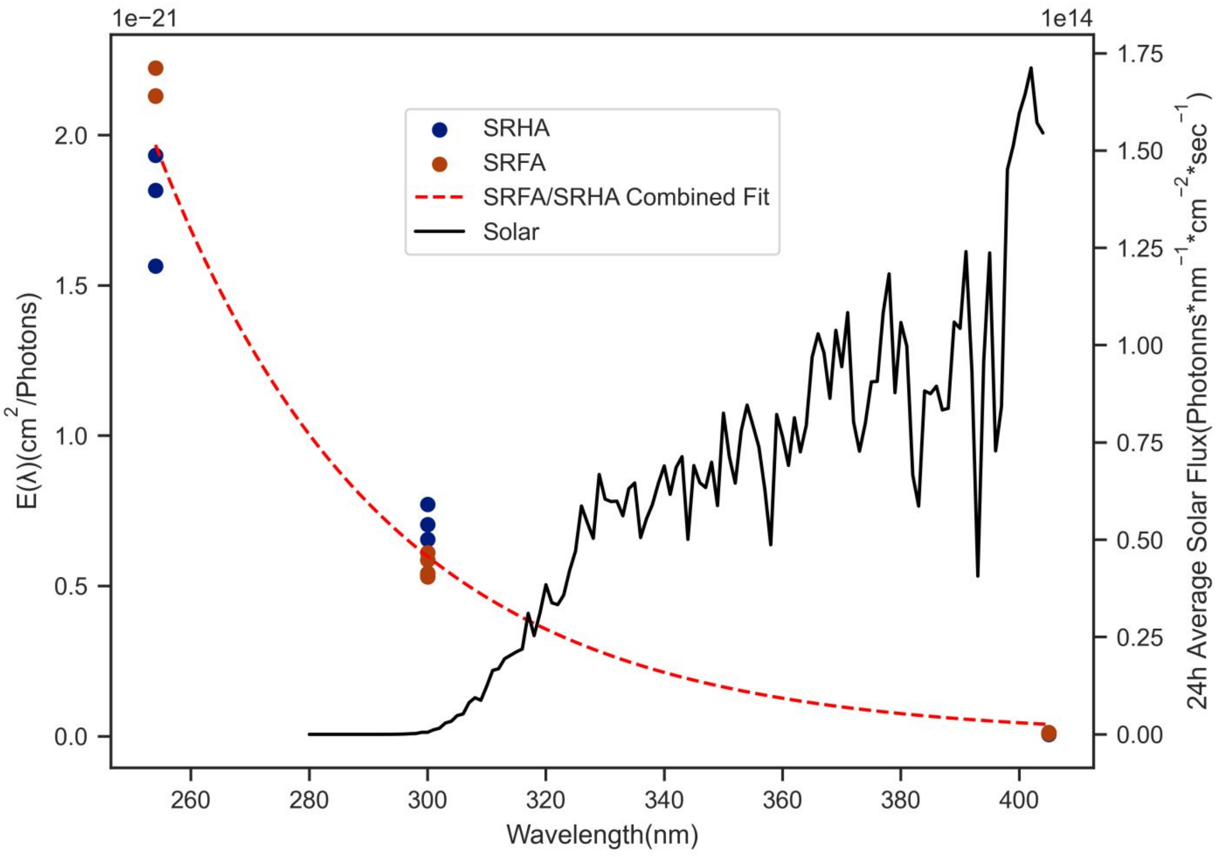

Figure S9. Exponential fit of fractional mass loss rate normalized to photon flux, E, (blue curve) and 24-hr average Athens, GA solar spectrum at 2023 summer solstice (06/21/2023) generated by the "Quick TUV" model.[3] Note that this spectrum has been averaged for 24 hours and therefore includes nightime hours (with no flux).

SRHA, SRFA data were combined and fit to a single exponential decay. The integral of the product of the exponential and the solar flux over the spectrum yields a fractional mass loss rate under atmospheric conditions, which is 8.25% mass lost over the first day in the atmosphere (during the summer solstice in Athens, GA in 2023). This rate is equivalent to 0.025% of J_{NO2} calculated for the same 24-hour period, including nighttime hours.

Parameters used to generate this solar spectrum in TUV model are shown below.

The following parameters were used to generate the solar spectrum:
-Latitude/Longitude: 33.95°N 83.357°W
-Date and Time: June 21, 2023, 00:00:00 GMT - June 21, 2023, 23:00:00 GMT
-Overhead Ozone: 300 du
-Surface Albedo: 0.1
-Ground Altitude: 0 km
-Measured Altitude: 0 km
-Clouds Optical Depth/Base/Top: 0:00/4.00/5.00
-Aerosols Optical Depth/S-S Albedo/Alpha: 0.235/0.990/1.000
-Sunlight Direct Beam/Diffuse Down/Diffuse Up: 1.0/1.0/0.0
-4 Streams Transfer Model

Figure S10. Laser Induced Ionization (LDI) HR-MS of SRFA sample

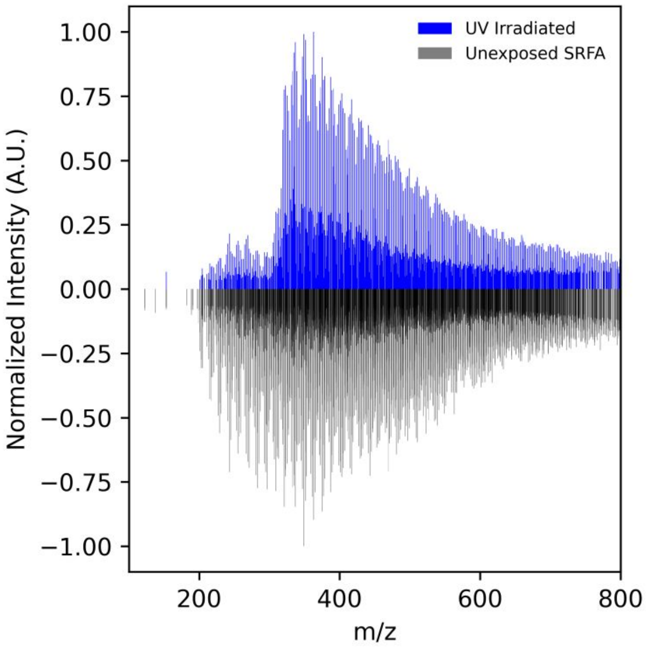

Figure S10. LDI-MS (laser desorption ionization-mass spectrometry) mass spectra of SRFA. The mass spectrum of UV-exposed SRFA sample is plotted with positive intensity (blue) while the mass spectrum of unexposed SRFA is plotted with negative intensity (black) for sake of comparison. Unlike the ESI condensed-phase photolysis experiments described in the main text, the LDI sample was exposed to the light directly on a MALDI plate. The LDI spectra shown here were only filtered by a signal cutoff of $3x10^5$ intensity level to remove noise. Formula assignment was not performed as the majority of peaks in LDI-MS cannot be assigned by MFAssignR due to its limitation with multiply-charged peaks. Comparing the irradiated sample to the raw SRFA material, a clear suppression of signals in the 100-300 m/z range was observed, which mirrors the results in the ESI-HR-MS spectra (Figure 7).

References

(1) Kawasaki, T.; Mochida, T.; Katada, J.-I.; Okahata, Y. *Laser Response of a Quartz Crystal Microbalance: Frequency Changes Induced by Light Irradiation in the Air Phase*; 2009; Vol. 25.

(2) Stanford Research Systems. *QCM100- Quartz Crystal Microbalance Theory and Calibration* . https://www.thinksrs.com/downloads/pdfs/applicationnotes/QCMTheoryapp.pdf (accessed 2024-02-24).

(3) Madronich, S. *ACOM: Quick TUV*. National Center for Atmospheric Research Atmospheric Chemistry Observations and Modeling. https://www.acom.ucar.edu/Models/TUV/Interactive_TUV/ (accessed 2024-01-02).

(4) Lignell, H.; Epstein, S. A.; Marvin, M. R.; Shemesh, D.; Gerber, B.; Nizkorodov, S. Experimental and Theoretical Study of Aqueous Cis-Pinonic Acid Photolysis. *J Phys Chem A* **2013**, *117* (48), 12930–12945. https://doi.org/10.1021/jp4093018.

(5) Bunce, N. J.; Lamarre, J.; Vaish, S. P. Photorearrangement of Azoxybenzene to 2-hydroxyazobenzene: a Convenient Chemical Actinometer. *Photochem Photobiol* **1984**, *39* (4), 531–533. https://doi.org/10.1111/j.1751-1097.1984.tb03888.x.
